# Onset of clinical and MRI efficacy occurs early after fingolimod treatment initiation in relapsing multiple sclerosis

**DOI:** 10.1007/s00415-015-7978-y

**Published:** 2015-12-08

**Authors:** Ludwig Kappos, Ernst-Wilhelm Radue, Peter Chin, Shannon Ritter, Davorka Tomic, Fred Lublin

**Affiliations:** 1 Neurology, Departments of Medicine, Clinical Research, Biomedicine and Biomedical Engineering, University Hospital, Petersgraben 4, 4031 Basel, Switzerland; 2 Medical Image Analysis Center, University Hospital Basel, Basel, Switzerland; 3 Novartis Pharmaceuticals Corporation, East Hanover, NJ USA; 4 Novartis Pharma AG, Basel, Switzerland; 5 Corinne Goldsmith Dickinson Center for Multiple Sclerosis, Icahn School of Medicine at Mount Sinai, New York, NY USA

**Keywords:** Brain volume loss, Cognition, Early treatment, Fingolimod, MRI, Multiple sclerosis, Relapse

## Abstract

To minimize the clinical burden associated with multiple sclerosis (MS), early control of focal and diffuse CNS disease activity is a treatment priority. A post hoc analysis was conducted to evaluate the onset of efficacy of fingolimod treatment in patients with relapsing MS. Data from patients who received fingolimod 0.5 mg or placebo during either of two 24-month, double-blind, randomized, parallel-group clinical trials (FREEDOMS and FREEDOMS II) were pooled for analysis. Efficacy outcomes were: time to first confirmed relapse; annualized relapse rate (ARR); proportions of patients free from T1 gadolinium-enhancing lesions or new/newly enlarged T2 lesions; percentage brain volume loss (BVL); and change in Multiple Sclerosis Functional Composite (MSFC) z-score from baseline to 6 months. An early benefit was seen with fingolimod (*N* = 783) vs. placebo (*N* = 773) for ARR at both 3 and 6 months (3 months, 0.32 vs. 0.52, *p* = 0.0015; 6 months, 0.21 vs. 0.45, *p* < 0.0001). Time to first relapse was also delayed with fingolimod vs. placebo from day 48 onwards. At 6 months, more patients in the fingolimod group than in the placebo group were free from new MRI activity (65.3 vs. 40.5 %, *p* < 0.0001) and had less BVL (37.1 % reduction vs. placebo, *p* < 0.001). MSFC z-score favored fingolimod over placebo at 6 months, with improvements noted in 9-Hole Peg Test and Paced Auditory Serial Addition Test scores. Improvements in outcomes related to relapses, MRI, disability, cognition, and BVL occurred within 6 months of treatment initiation with fingolimod.

**Electronic supplementary material:**

The online version of this article (doi:10.1007/s00415-015-7978-y) contains supplementary material, which is available to authorized users.

## Introduction

Early reduction of disease activity is an important therapeutic goal for patients with multiple sclerosis (MS) to minimize neuro-axonal damage, prevent irreversible accumulation of disability and prolong survival [1, 10]. The initial phase 2 study of oral fingolimod (FTY720; Gilenya^®^, Novartis Pharma AG, Basel, Switzerland) in patients with relapsing MS showed reductions in inflammatory activity evident on magnetic resonance imaging (MRI) as early as 2 months into treatment; reductions in annualized relapse rate (ARR) were reported within 6 months, albeit using higher doses of fingolimod than the approved, once-daily 0.5 mg dose [12]. At this lower dose, fingolimod significantly reduced clinical and MRI disease activity compared with interferon β-1a i.m. [5] and placebo [2, 15] in phase 3 studies over 12 and 24 months, with effects on MRI outcomes evident within 6 months. Brain volume loss (BVL), which can occur in the earliest stages of MS as a consequence of focal inflammatory and diffuse damage to the central nervous system (CNS) [1], was also significantly reduced by fingolimod in the first 6 months of therapy [12, 17]. Within the same time frame, fingolimod reduced the conversion of baseline T1 gadolinium (Gd)-enhancing MRI lesions into black holes, indicative of decreased permanent damage in the brain [16].

Using a pooled population from the two placebo-controlled, phase 3 studies (FTY720 Research Evaluating Effects of Daily Oral Therapy in MS [FREEDOMS; ClinicalTrials.gov number, NCT00289978] and FREEDOMS II [ClinicalTrials.gov number, NCT00355134]), clinical and MRI measures were assessed to establish the timing of the onset of treatment effects during the first 6 months of fingolimod therapy.

## Materials and methods

### Patients and study design

The study design and overall results for FREEDOMS and FREEDOMS II have been reported previously [2, 15]. In brief, FREEDOMS and FREEDOMS II were 24-month, double-blind, randomized, parallel-group clinical trials comparing the efficacy and safety of two oral doses of fingolimod (0.5 and 1.25 mg/day) with placebo in patients 18–55 years of age with active relapsing–remitting MS (RRMS).

In both trials, standardized MRI scans were performed at screening, 6, 12, and 24 months after initiation of treatment. Multiple Sclerosis Functional Composite (MSFC) z-scores were determined at baseline and at 6-month intervals thereafter. The same definition of a confirmed relapse was applied in both FREEDOMS and FREEDOMS II: symptoms were required to be accompanied by an increase of at least half a point in the Expanded Disability Status Scale (EDSS) score, or of one point in the score for two different functional systems of the EDSS, or of two points in the score for one of the functional systems (excluding bowel, bladder, or cerebral functional systems).

### Statistical analyses

Pooled data from FREEDOMS and FREEDOMS II were analyzed post hoc for treatment differences between the fingolimod 0.5 mg and placebo groups in relapse and MRI endpoints within the first 6 months. The time to first confirmed relapse was estimated using the Kaplan–Meier method. The effect of fingolimod and placebo on time to first relapse was compared using a log-rank test. ARRs in the two treatment arms were compared using a Poisson regression model, adjusted for treatment, study, number of relapses in the 2 years before enrollment, and core baseline EDSS score; log(time in study) was the offset variable.

The proportions of patients free from T1 Gd-enhancing lesions or new/newly enlarged T2 lesions were analyzed using a logistic regression model adjusted for treatment, study, pooled country, and corresponding MRI baseline measurement. Percentage brain volume change (PBVC) from baseline [determined using Structural Image Evaluation using Normalization of Atrophy (SIENA) methodology as a] measure of BVL was compared between treatment arms using rank analysis of covariance (ANCOVA; adjusted for treatment, study, pooled country, and baseline normalized brain volume). Change from baseline in MSFC z-score to 6 months was compared between treatment arms using rank ANCOVA (adjusted for treatment, study, the corresponding baseline value, and age). Analyses were conducted in the pooled intent-to-treat population (full analysis set), without multiplicity adjustments.

## Results

### Study population

Of the 2355 patients in the pooled population of FREEDOMS and FREEDOMS II, 783 were randomized to receive fingolimod 0.5 mg and 773 were randomized to the placebo group. Baseline demographic and clinical characteristics of patients in the two individual studies have been reported previously [2, 15] and were generally similar. The pooled study population was consistent with a typical population of patients with active RRMS (Online Resource 1).

### Early effects of treatment on clinical outcomes

**Fig. 1:**
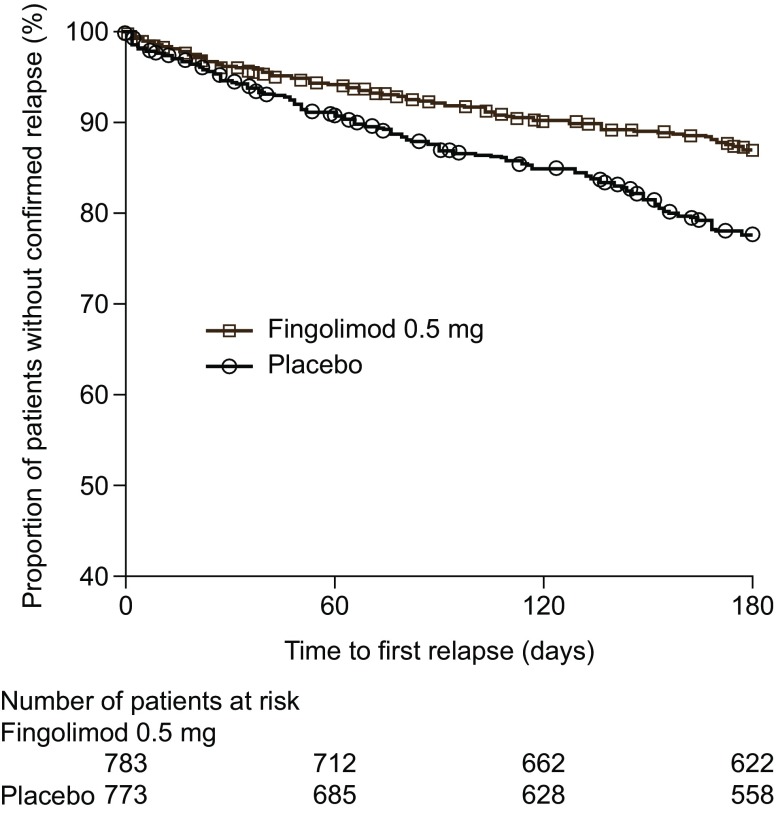
Time to first confirmed MS relapse in the pooled FREEDOMS and FREEDOMS II population (intent-to-treat populations). A delay in the time to first confirmed MS relapse was first observed on day 48 (*p* ≤ 0.05; log-rank test) in the pooled FREEDOMS and FREEDOMS II population

At 3 months, fingolimod reduced ARR compared with placebo (38.5 % reduction, *p* = 0.0015); this treatment effect was maintained over months 3–6 (53.3 % reduction, *p* < 0.0001; Table 1). The difference in time to first confirmed relapse between the fingolimod 0.5 mg and placebo groups reached significance (*p* ≤ 0.05) at day 48 and remained significant thereafter (Fig. 1). Based on Kaplan–Meier estimates, the proportion of patients free from confirmed relapses was significantly higher with fingolimod than with placebo at 3 and 6 months, equating to reductions of 35.5 and 42.8 %, respectively, in the risk of having confirmed relapse (Table 1). The change from baseline to 6 months in MSFC z-score favored fingolimod over placebo [mean (median): −0.01 (0.02) vs. −0.04 (−0.04), respectively; *p* < 0.0001; Table 1]. Similarly, compared with placebo, fingolimod improved the outcome for two of the three individual MSFC subscales [Paced Auditory Serial Addition Test (PASAT) and 9-Hole Peg Test] (Table 1).

**Table 1:** Clinical measures of disease activity in the first 6 months after initiation of fingolimod therapy in the pooled FREEDOMS and FREEDOMS II population

	Fingolimod 0.5 mg *N* = 783	Placebo *N* = 773	Fingolimod 0.5 mg *N* = 783	Placebo *N* = 773
Patients free from confirmed relapse	At 3 months		At 6 months	
Number (%) of patients free from confirmed relapse	717 (91.6)	670 (86.7)	681 (87.0)	598 (77.4)
Kaplan–Meier estimate of patients free from confirmed relapse, % ± SE (95 % CI)	91.4 ± 1.0 (89.4, 93.4)	86.4 ± 1.3 (84.0, 88.9)	86.4 ± 1.3 (84.0, 88.9)	76.5 ± 1.6 (73.5, 79.6)
*p* value vs. placebo^a^	0.0022		< 0.0001	
Hazard ratio for fingolimod vs. placebo (95 % CI)^b^	0.64 (0.47, 0.88) *p* = 0.0056		0.57 (0.45, 0.73) *p* < 0.0001	
Annualized relapse rate	Months 0–3		Months 3–6	
Number of patients	783	773	766	754
ARR (95 % CI)	0.32 (0.25, 0.41)	0.52 (0.43, 0.63)	0.21 (0.16, 0.28)	0.45 (0.37, 0.55)
Rate ratio vs. placebo (95 % CI)^c^	0.61 (0.45, 0.83) *p* = 0.0015		0.47 (0.33, 0.66) *p* < 0.0001	
MSFC z-score	Months 0–3		Months 0–6	
Baseline (mean ± SD)	–	–	0.08 ± 0.71	−0.03 ± 0.92
Change from baseline				
Mean ± SD	–	–	−0.01 ± 0.47	−0.04 ± 0.63
Median (range)	–	–	0.02 (−6.3 to 3.1)	−0.04 (−3.2 to 9.7)
*p* value vs. placebo^d^	–	–	< 0.0001	
MSFC subscale: T25FW				
Baseline (mean ± SD) (s)	–	–	5.67 ± 2.64	6.09 ± 5.18
Change from baseline (s)				
Mean ± SD	–	–	0.16 ± 3.00	0.01 ± 4.49
Median (range)	–	–	0.00 (−19.5 to 52.7)	0.10 (−82.6 to 18.6)
*p* value vs. placebo^d^	–	–	0.0032	
MSFC subscale: 9-HPT				
Baseline (mean ± SD) (s)	–	–	21.80 ± 6.20	22.23 ± 6.90
Change from baseline (s)				
Mean ± SD	–	–	0.35 ± 5.68	1.04 ± 12.54
Median (range)	–	–	0.05 (−32.6 to 89.4)	0.28 (−24.3 to 289.1)
*p* value vs. placebo^d^	–	–	0.0041	
MSFC subscale: PASAT				
Baseline (mean ± SD) (number of correct answers)	–	–	48.6 ± 10.26	47.4 ± 11.07
Change from baseline (number of correct answers)				
Mean ± SD	–	–	0.6 ± 5.93	−0.2 ± 6.43
Median (range)	–	–	0 (−27 to 34)	0 (−47 to 35)
*p* value vs. placebo^d^	–	–	0.0146	

For MRI data, percentages were calculated using the number of patients with an evaluable MRI scan as denominator: 727 and 702 (Gd-enhancing T1 lesions) and 732 and 723 (new/newly enlarged T2 lesions) patients in the fingolimod 0.5 mg pooled group and placebo pooled group, respectively. For patients free from new MRI activity, the denominator was the same as for the fingolimod 0.5 mg pooled group. The means and medians were calculated on the basis of all images, not just those showing lesions

*ARR* annualized relapse rate, *CI* confidence interval, *9-HPT* 9-Hole Peg Test, *T25FW* Timed 25-Foot Walking Test, *Gd* gadolinium, *MSFC* Multiple Sclerosis Functional Composite, *PASAT* Paced Auditory Serial Addition Test, *PBVC* percent brain volume change, *SE* standard error

^a^ *p* values for treatment comparison were based on a log-rank test using day 104, 194, 374, and 734 as the cutoff for censoring at month 3, 6, 12, and 24, respectively

^b^Hazard ratios were derived from a Cox’s proportional hazards model adjusted for treatment, study, pooled country, country or region, baseline number of relapses in the 2 years before enrollment, and baseline EDSS score

^c^ *p* values for treatment comparison were from a Poisson regression model, adjusted for treatment, study, number of relapses in the 2 years before enrollment, and core baseline EDSS score; log(time in study) was the offset variable

^d^ *p* value calculated using rank analysis of covariance adjusted for treatment, study, the corresponding baseline value, and age

### Early effects of treatment on MRI outcomes

Compared with placebo, fingolimod reduced the number of Gd-enhancing T1 lesions by 83.8 % and new/newly enlarged T2 lesions by 72.6 % over 6 months (first on-study MRI) (Table 2). Similarly, a significantly greater proportion of patients was free from Gd-enhancing T1 lesions (42.0 % increase) and also free from new/newly enlarged T2 lesions (63.3 % increase) at 6 months in the fingolimod group than in the placebo group (Table 2). At 6 months, the proportion of patients free from any new MRI activity was significantly higher in the fingolimod group than in the placebo group (61.2 % increase; Table 2). A significant difference in the PBVC was seen between the fingolimod group and the placebo group at 6 months, with 37.1 % less BVL evident in the fingolimod group (Table 2).

**Table 2:** MRI measures of disease activity in the first 6 months after initiation of fingolimod therapy in the pooled FREEDOMS and FREEDOMS II population

	Fingolimod 0.5 mg *N* = 783	Placebo *N* = 773
Number of Gd-enhancing T1 lesions	6 months	
Number of patients	726	698
Mean ± SD	0.2 ± 0.9	1.2 ± 3.2
Median (range)	0.0 (0–13)	0.0 (0–43)
Number (%) of patients free from Gd-enhancing T1 lesions	644 (88.6)	438 (62.4)
*p* value vs. placebo^a^	<0.0001	
Number of new/newly enlarged T2 lesions	Months 0–6	
Number of patients	729	721
Mean ± SD	0.9 ± 2.3	3.3 ± 7.0
Median (range)	0.0 (0–28)	1.0 (0–96)
Number (%) of patients free from new/newly enlarged T2 lesions	478 (65.3)	289 (40.0)
*p* value vs. placebo^b^	<0.0001	
Patients free from new MRI activity	At 6 months	
Number (%) of patients free from new MRI activity^c^	475 (65.3)	284 (40.5)
*p* value vs. placebo^a^	<0.0001	
Brain volume loss	At 6 months	
Number of patients	714	709
Mean PBVC from baseline	−0.23	−0.36
Reduction vs. placebo (%)	37.1	
*p* value vs. placebo^d^	<0.001	

For MRI data, percentages were calculated using the number of patients with an evaluable MRI scan as denominator: 727 and 702 (Gd-enhancing T1 lesions) and 732 and 723 (new/newly enlarged T2 lesions) patients in the fingolimod 0.5 mg pooled group and placebo pooled group, respectively. For patients free from new MRI activity, the denominator was the same as for the fingolimod 0.5 mg pooled group. The means and medians were calculated on the basis of all images, not just those showing lesions

*Gd* gadolinium, *MRI* magnetic resonance imaging, *PBVC* percentage brain volume change

^a^ *p* value calculated using a logistic regression model adjusted for treatment, study, pooled country, and baseline number of Gd-enhancing T1 lesions

^b^ *p* value calculated using a logistic regression model adjusted for treatment, study, and pooled country

^c^Patients free from new MRI activity are patients who have no Gd-enhancing T1 lesions and no new/newly enlarged T2 lesions

^d^ *p* values are from rank analysis of covariance adjusted for treatment, study, pooled country, and baseline normalized brain volume, and indicate two-sided significance at the 0.05 level

## Discussion

In patients with active MS, early initiation of, and adherence to, a disease-modifying therapy (DMT) that rapidly controls disease activity is important to minimize acute inflammation and its neuropathological sequelae, and also to prevent subsequent disease activity. Furthermore, treating patients with MS early in the disease course with agents that not only target relapses but also subclinical, silent disease (including BVL) could provide long-term benefits. The current analyses of pooled data from the phase 3, placebo-controlled studies indicate that the onset of action of fingolimod on relapses, MRI lesions, BVL, upper extremity function, and cognition commenced early, within 3–6 months of treatment initiation. The early effect on relapses and MRI lesions is consistent with the results obtained in the phase 2 study: once-daily fingolimod 1.25 and 5.0 mg increased the proportion of patients who were relapse-free over 6 months and free from Gd-enhancing T1 lesions as early as 2 months after therapy initiation. In FIRST (Fingolimod Initiation and caRdiac Safety Trial), an effect on relapses was seen within 2–4 months of starting therapy with the approved dose of 0.5 mg fingolimod, irrespective of patients’ previous treatment experience [6, 7, 12].

Among the most salient evidence of an early treatment effect of fingolimod was the reduction in the rate of BVL within the first 6 months, seen here with the pooled population and reported previously in the individual studies [2, 15, 17]. This effect may be related to preclinical and in vitro findings of direct effects of fingolimod on the CNS [3], and is further substantiated by the effect of fingolimod, in decreasing the evolution of inflammatory lesions into black holes, as seen at 6 months in the FREEDOMS study [16].

Early treatment effects have been reported for other approved DMTs, with improvements in relapse rates at 3 months with natalizumab and dimethyl fumarate (DMF), in a composite measure of MRI lesions at week 4 with interferon β-1a, and in overall disease activity at 6 months (clinical and MRI composite) with DMF [9, 11, 13, 14]. However, none of these treatments had an effect at 6 months on a clinical measure of disability or on BVL [9, 11, 13, 14]; any reported effects on brain atrophy were delayed beyond the first year of therapy, this delay usually being attributed to “pseudoatrophy” caused by an anti-inflammatory effect of DMTs occurring within the first year of therapy [8]. Notably, the significant reduction in BVL observed with fingolimod at 6 months was achieved despite a pronounced and early reduction in inflammatory activity. Taken together, the effects of fingolimod on BVL, deep grey matter [18] and MSFC outcomes, including the PASAT cognition subscale score [4], suggest that fingolimod may also modify correlates of diffuse CNS damage early after the initiation of treatment.

The limitations of these post hoc analyses include the lack of adjustment for multiplicity and, owing to the low number of relapses up to month 6 (compared up to months 12 and 24), the use of a Poisson model for ARR analysis rather than the negative binomial model used in the pivotal study analysis; however, these limitations should be weighed against the large number of patients in the pooled population. In addition, it should be recognized that the PASAT is not a global measure of cognitive function, and suffers from marked practice effects [19], although in FREEDOMS and FREEDOMS II, the impact of practice effects should have been reduced by patients undertaking three PASAT training sessions during the pre-treatment period.

Overall, the analyses reported here indicate that, within 6 months of initiation, treatment benefits of fingolimod were evident on key measures of focal and diffuse disease in relapsing MS, i.e., relapses, MRI lesions and BVL, as well as on elements of disability and cognitive function.

## Electronic supplementary material

Below is the link to the electronic supplementary material.

Supplementary material 1 (DOC 80 kb)
