# Supplementary material for: Onset of clinical and MRI efficacy occurs early after fingolimod treatment initiation in relapsing multiple sclerosis
Source: J Neurol. 2015 Dec 8;263(2):354–60. doi: 10.1007/s00415-015-7978-y (PMC4751181; doi:10.1007/s00415-015-7978-y)
Supplement: Supplementary file 1 — Supplementary material 1 (DOC 80 kb) [file 415_2015_7978_MOESM1_ESM.doc]

**Online Resource 1. Baseline demographics and disease characteristics of the pooled FREEDOMS and FREEDOMS II population (full analysis set)**

| **Characteristic** | **Fingolimod 1.25 mg (*N* = 799)** | **Fingolimod 0.5 mg (*N* = 783)** | **Placebo  (*N* = 773)** | **Total  (*N* = 2355)** |
| --- | --- | --- | --- | --- |
| Age, years  Mean ± SD  Median (range) | 39.0 ± 9.1  40 (17–57) | 38.4 ± 8.8  38 (18–55) | 38.6 ± 8.6  39 (18–55) | 38.6 ± 8.8  39 (17–57) |
| Women, *N* (%) | 576 (72.1) | 571 (72.9) | 586 (75.8) | 1733 (73.6) |
| Time (years) from first MS symptom to randomization  Mean ± SD  Median (range) | 9.5 ± 7.6  7.8 (0–50) | 9.1 ± 7.4  7.2 (0–49) | 9.3 ± 7.2  7.8 (0–40) | 9.3 ± 7.4  7.6 (0–50) |
| Relapses within previous year, *N*  Mean ± SD  Median (range) | 1.5 ± 0.9  1.0 (0–12) | 1.4 ± 0.8  1.0 (0–6) | 1.5 ± 0.8  1.0 (0–7) | 1.5 ± 0.8  1.0 (0–12) |
| Relapses within previous 2 years, *N*  Mean ± SD  Median (range) | 2.2 ± 1.7  2.0 (1–30) | 2.2 ± 1.2  2.0 (1–11) | 2.2 ± 1.3  2.0 (1–14) | 2.2 ± 1.4  2.0 (1–30) |
| EDSS score  Mean ± SD  Median (range) | 2.5 ± 1.3  2.5 (0–6.0) | 2.3 ± 1.3  2.0 (0–6.5) | 2.5 ± 1.3  2.0 (0–6.0) | 2.4 ± 1.3  2.0 (0–6.5) |
| No history of disease-modifying treatment, *N* (%) | 342 (42.8) | 338 (43.2) | 345 (44.6) | 1025 (43.5) |
| Absence of Gd-enhancing T1 lesions, *N* (%) | 511 (64.6) | 481 (61.6) | 487 (63.2) | 1479 (63.2) |
| Number of Gd-enhancing lesions on T1-weighted images  Mean ± SD  Median (range) | 1.6 ± 4.2  0 (0–50) | 1.5 ± 4.7  0 (0–84) | 1.2 ± 3.1  0 (0–46) | 1.4 ± 4.1  0 (0–84) |
| Volume of lesions on T2-weighted images, mm^3^  Mean ± SD  Median (range) | 5952 ± 8006  2838 (0–55,527) | 5833 ± 7799  2964 (0–54,369) | 5882 ± 7443  3074 (0–69,203) | 5890 ± 7752  2941 (0–69,203) |
| Volume of hypointense lesions on T1-weighted images, mm^3^  Mean ± SD  Median (range) | 1664 ± 2875  534 (0–25,886) | 1678 ± 2935  552 (0–23,937) | 1719 ± 2964  555 (0–20,956) | 1687 ± 2732  549 (0–25,886) |
| Normalized bran volume, cc  Mean ± SD  Median (range) | 1514 ± 83  1516 (1217–1764) | 1521 ± 83  1529 (1144–1734) | 1519 ± 86  1522 (1230–1756) | 1518 ± 84  1522 (1144–1764) |

EDSS, Expanded Disability Status Scale; Gd, gadolinium; MRI, magnetic resonance imaging; MS, multiple sclerosis; SD, standard deviation

For MRI data (absence of Gd-enhancing T1 lesions), percentages were calculated using the number of patients with an evaluable MRI scan as denominator: 791 patients in the fingolimod 1.25 mg pooled group, 781 patients in the fingolimod 0.5 mg pooled group, 770 patients in the placebo pooled group, and 2342 patients in the total pooled population. The means and medians were calculated on the basis of all images, not just those showing lesions
